# Dysregulation of Mir-196b in Head and Neck Cancers Leads to Pleiotropic Effects in the Tumor Cells and Surrounding Stromal Fibroblasts

**DOI:** 10.1038/s41598-017-18138-8

**Published:** 2017-12-19

**Authors:** Saúl Álvarez-Teijeiro, Sofía T. Menéndez, M. Ángeles Villaronga, Juan P. Rodrigo, Lorea Manterola, Lucas de Villalaín, Juan C. de Vicente, Laura Alonso-Durán, M. Pilar Fernández, Charles H. Lawrie, Juana M. García-Pedrero

**Affiliations:** 1 Department of Otolaryngology, Hospital Universitario Central de Asturias and Instituto de Investigación Sanitaria del Principado de Asturias, IUOPA, University of Oviedo, Oviedo, CIBERONC Spain; 2 Molecular Oncology Group, Biodonostia Research Institute, San Sebastián, Spain; 3 Department of Oral and Maxillofacial Surgery, Hospital Universitario Central de Asturias and Instituto de Investigación Sanitaria del Principado de Asturias, IUOPA, University of Oviedo, Oviedo, Spain; 4 Department of Biochemistry and Molecular Biology, University of Oviedo, Oviedo, Spain; 5 IKERBASQUE, Basque Foundation for Science, Bilbao, Spain

## Abstract

The miR-196 family members have been found dysregulated in different cancers. Therefore, they have been proposed as promising biomarkers and therapeutic targets. This study is the first to investigate the role of miR-196b in the development and progression of head and neck squamous cell carcinomas (HNSCC), and also the impact on the surrounding tumor microenvironment. Increased miR-196b levels were detected in 95% of primary tumors and precancerous lesions, although no significant differences were observed between non-progressing *versus* progressing dysplasias. Furthermore, increased levels of both miR-196a and miR-196b were successfully detected in saliva samples from HNSCC patients. The functional consequences of altered miR-196 expression were investigated in both HNSCC cell lines and cancer-associated fibroblasts (CAFs) by transfection with specific pre-miR precursors. Results showed that both miR-196a and miR-196b elicit cell-specific responses in target genes and downstream regulatory pathways, and have a distinctive impact on cell proliferation, migration and invasion. These data reveal the early occurrence and prevalence of miR-196b dysregulation in HNSCC tumorigenesis, suggesting its utility for early diagnosis and/or disease surveillance and also as a non-invasive biomarker in saliva. The pleiotropic effects of miR-196a/b in HNSCC cell subpopulations and surrounding CAFs may complicate a possible therapeutic application.

## Introduction

Head and neck squamous cell carcinoma (HNSCC) is a highly heterogeneous disease in terms of etiology, with multiple genetic and molecular alterations and varying biologic behavior and clinical outcomes^1,2^. Therefore much work has focused on the identification of biologic and molecular factors that may serve as prognostic and predictive markers, as well as new targets for therapy^3^.

MicroRNAs (miRNAs) have emerged as crucial players^4–7^ in regulating gene expression in normal homeostasis and also numerous disorders, including autoimmune, cardiovascular and neurodegenerative diseases and cancer^8–10^.

The members of the miR-196 gene family (miR-196a-1, miR-196a-2, and miR-196b) are transcribed from three different genes, which are located in homeobox (HOX) gene cluster regions in humans^11,12^. The mature nucleotide sequences of miR-196a-1 of miR-196a-2 are identical, whereas mature miR-196b differs from miR-196a by one nucleotide^12^. Numerous studies indicate that the miR-196 family plays critical roles in normal development and pathogenesis, and may therefore have great potential in clinical applications such as the diagnosis, prognosis and/or treatment of developmental defects, viral infections and different cancers^13,14^. miR-196a has been found upregulated in numerous cancer types, and proven to have pro-oncogenic functions promoting cell proliferation, migration, invasion and radioresistance^15–21^. miR-196b upregulation has been described in oral, gastric, colorectal cancer, glioblastoma and leukemia^22–25^. However, some reports have also shown that the miR-196 family members may act as tumor suppressors. Thus, miR-196a suppressed metastasis in melanoma and breast cancers^25,26^, and miR-196b down-regulation has been reported in different types of leukemia^27–30^. Even though the frequent dysregulation of miR-196 family in cancer indicates a close association with disease status, the clinical and biological significance and regulatory networks have been explored to a very limited extent.

This study is the first to investigate the pathobiological role and clinical relevance of miR-196b in HNSCC and the possible influence on the surrounding tumor microenvironment. This was accomplished by monitoring miRNA expression in HNSCC tissue specimens, HNSCC-derived cell lines and cancer-associated fibroblasts (CAFs) isolated from HNSCC patients. The present study also explored for the first time the role of miR-196b in early stages of HNSCC tumorigenesis and malignant transformation by analyzing miRNA expression in a large series of premalignant lesions, seeking possible correlations with the risk of progression to invasive carcinoma. In addition, the abundance and potential utility of miR-196a/b as minimally invasive biomarkers were assessed in saliva samples. *In vitro* functional analysis demonstrated that altered miR-196a/b expression causes pleiotropic effects in HNSCC and the surrounding microenvironment, thereby eliciting cell-specific responses in target genes and downstream regulatory pathways, with distinctive impact on cell proliferation, migration and invasion.

## Results

### Frequent dysregulation of miR-196b expression in HNSCC tissue specimens

**Figure 1:**
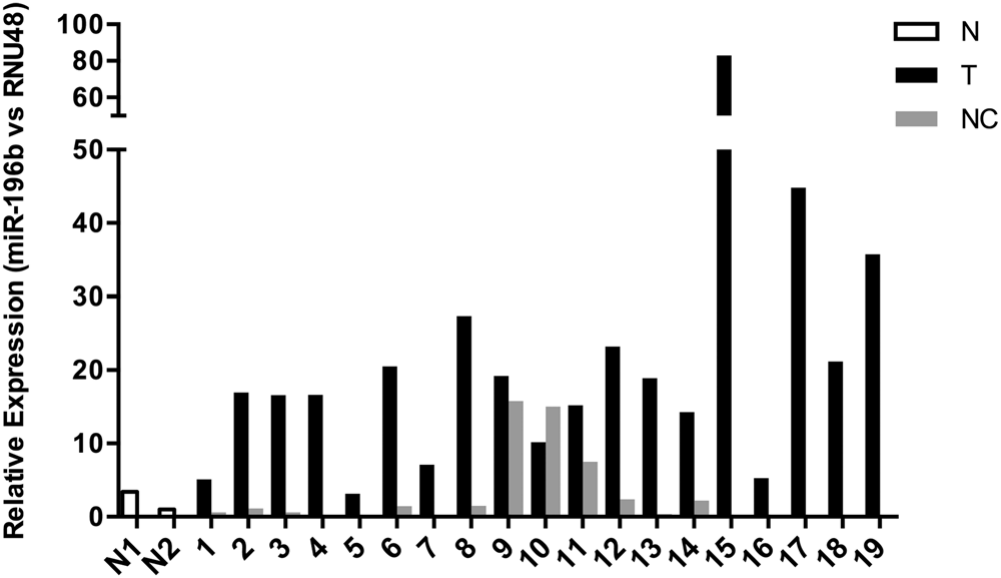
Analysis of miR-196b expression in HNSCC tissue specimens. miR-196b expression levels were quantified by RT-qPCR in 19 fresh primary tumors (T) and patient-matched normal counterparts (NC). Data were normalized to RNU48 levels, and relative to the normal mucosa from non-oncologic patients (N).

miR-196 expression levels were analyzed using Taqman™ miRNA assays in 19 fresh HNSCC tissue specimens and 11 patient-matched normal epithelia. miR-196b levels were found to significantly increase in 18 (95%) tumors (T) (range 4 to 83-fold) and also in 3 (27%) patient-matched normal mucosa (NC) (range 7 to 15-fold), compared to non-oncologic patients (N) (Fig. 1). miR196a levels were also found to frequently increase in HNSCC tissue specimens, in accordance to previous reports^14–16^. Thus, increased miR-196a levels were detected in 100% of tumors (range 3 to 159-fold) and also in 5 of 11 (45%) patient-matched normal epithelia (range 3 to 18-fold) (Supplementary Figure S1).

We next assessed potential associations between miR-196a and miR-196b expression and clinicopathologic characteristics (Supplementary Figure S2). However, no significant differences were observed when comparing miR-196a and miR-196b levels by tumor site (larynx *versus* pharynx), lymph node infiltration (pN classification, dichotomized as N0 *versus* N1–3), and disease stage (IV *versus* I–III).

### Expression of miR-196b in early stages of HNSCC tumorigenesis

miR-196b expression was evaluated in formalin-fixed paraffin-embedded (FFPE) tissue samples from 40 patients with laryngeal dysplasia, comprising 17 non-progressing and 23 progressing lesions. miRNA expression was also analyzed in the 23 patient-matched invasive tumors subsequently developed and compared to that of the corresponding premalignant lesion. Normal epithelium (FFPE) obtained from 5 non-oncologic patients without exposure to tobacco carcinogens was used as healthy control.

**Figure 2:**
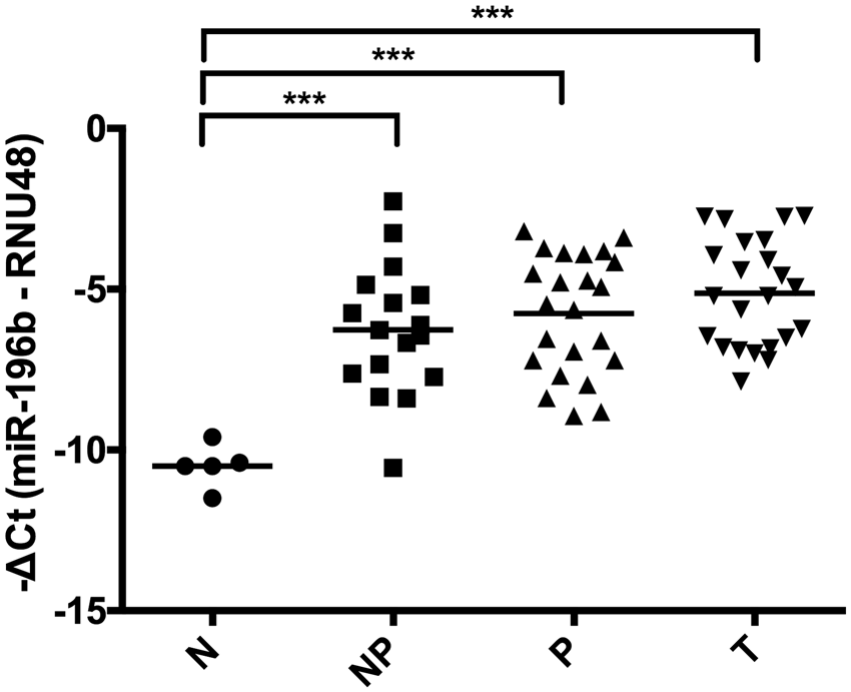
Analysis of miR-196b expression in early stages of HNSCC tumorigenesis. miR-196b expression levels were quantified by RT-qPCR in 40 patients with laryngeal precancerous lesions, comprising 17 non-progressing dysplasias (NP), 23 progressing dysplasias (P) and the 23 patient-matched invasive tumors (T) subsequently developed. Normal epithelia from 5 non-oncologic patients were included as (N) healthy controls. ***p < 0.001 by Holm-Sidak’s multiple comparisons test.

Quantitative analysis showed that significantly increased levels of miR-196b were detected in an overwhelmingly high percentage of precancerous lesions (95%), compared to healthy controls (Fig. 2). These data documented the early occurrence and high prevalence of miR-196b dysregulation in HNSCC tumorigenesis; however, no significant differences were observed when comparing the expression levels in non-progressing *versus* progressing dysplasias and patient-matched tumors subsequently developed. Similar results were obtained from the analysis of miR196a levels using the same set of precancerous lesions (Supplementary Figure S3).

### Detection of miR-196a and miR-196b expression in saliva samples from HNSCC patients

**Figure 3:**
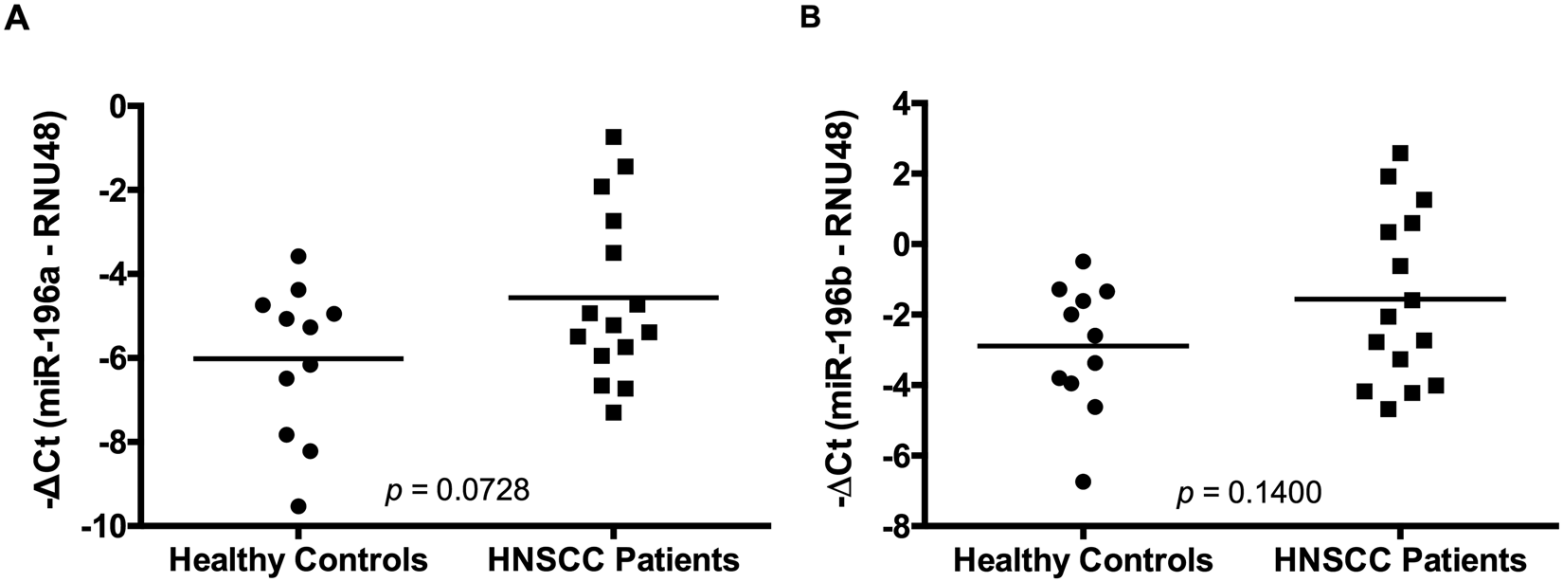
Analysis of miR-196a and miR-196b expression in saliva samples. **(A**) miR-196a and (**B**) miR-196b expression levels were quantified by RT-qPCR in saliva samples from 15 HNSCC patients and 11 healthy controls and normalized to RNU48 levels with *p* values calculated by Student t-test.

The frequent miR-196a/b dysregulation in HNSCC patients (95–100%) prompted us to investigate the abundance and potential utility of both miRNAs as minimally invasive biomarkers for the early diagnosis and/or monitoring of disease status. miR-196a and miR-196b expression was successfully detected in all saliva samples tested (from 15 HNSCC patients and 11 healthy controls). Furthermore, increased levels of both miRNAs were observed in saliva samples from HNSCC patients compared to healthy controls (Fig. 3), with trends almost reaching statistical significance.

### *In vitro* functional role of miR-196a and miR-196b expression in HNSCC-derived cell lines and cancer-associated fibroblasts

There is a crosstalk between cancer cells and the surrounding stroma. miRNAs may play an important role as they can be secreted from tumor cells into exosomes, thereby modulating the tumor microenvironment acting as paracrine signaling factors. Consequently, the pathobiological role of altered expression of miR-196a and miR-196b in HNSCC was assessed in both HNSCC-derived cell lines and cancer-associated fibroblasts (CAFs), which are a major constituent of the tumor stroma. A series of functional studies were carried out using various *in vitro* cellular models (FaDu, UT-SCC-42B, and CAFs) transiently transfected with specific pre-miR precursors to determine their impact on cell proliferation, migration and invasion. Transfection with pre-miR-196a and pre-miR-196b respectively led to a robust induction of mature miR-196a and miR-196b levels in HNSCC cells and CAFs, compared to the endogenous levels in control-transfected cells (Supplementary Figure S4). Although some cross-reactivity was seen between pre-miR precursors and mature miR-196a and miR-196b, specificity was >90% in all cases.

**Figure 4:**
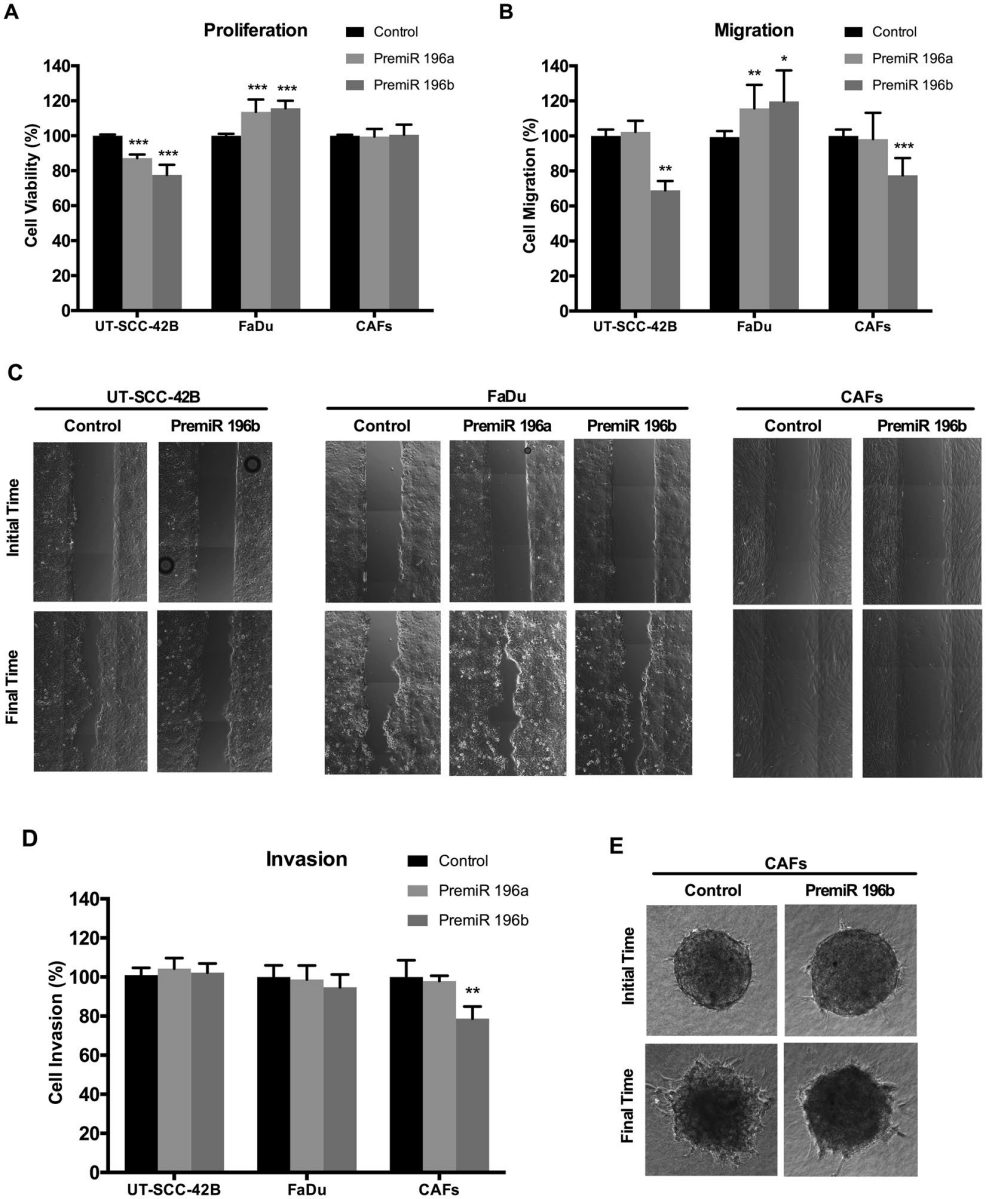
Functional analysis of miR-196a and miR-196b in HNSCC-derived cell lines and CAFs. UT-SCC-42B, FaDu cells and CAFs were transfected with either premiR-196a, premiR-196b or non-targeting control, as indicated. (**A**) Cell proliferation was estimated by Tetrazolium-based MTS assay after 4 days. Data were normalized to the absorbance at day 0 and relative to control-transfected cells. (**B**) Bar chart showing the relative migration of UT-SCC-42B, FaDu or CAFs transfected with premiR-196a, premiR-196b or non-targeting control. (**C**) Representative images from the wound healing assays showing the initial scratch (t = 0) area and the residual area at each established final time (5, 15 or 20 h for UT-SCC-42B, FaDu or CAFs, respectively). (**D**) Bar chart showing the relative invasion of UT-SCC-42B, FaDu or CAFs transfected with premiR-196a, premiR-196b or non-targeting control. (**E**) Representative images from the 3D invasion assays of CAFs spheroids embedded into a collagen matrix. The invasive area was determined by calculating the difference between the final area (t = 20 h) and the initial area (t = 0) using Image J analysis program. All data were normalized to control-transfected cells and expressed as the mean ± SD of at least three independent experiments performed in quadruplicate. *p < 0.05, **p < 0.01 and ***p < 0.001 by Student t-test.

The functional consequences of miR-196a and miR-196b over-expression varied depending on the cell type. Thus, cell proliferation significantly increased in FaDu cells transfected with pre-miR196a or pre-miR196b compared to control cells, whereas proliferation significantly decreased upon pre-miR transfection in UT-SCC-42B cells or remained unchanged in CAFs (Fig. 4A). Similarly, transfection with either pre-miR-196a or pre-miR-196b also significantly increased migration in FaDu cells, while transfection with pre-miR-196b specifically decreased cell migration in both UT-SCC-42B and CAFs (Fig. 4B and C). In addition, transfection with pre-miR-196b specifically decreased cell invasion in CAFs, whereas it had no effect in the two HNSCC cell lines tested (Fig. 4D–E). These data evidence that altered expression of miR-196a and miR-196b causes pleiotropic effects depending on specific cellular and tissue contexts.

### Identification of endogenous target genes in HNSCC-derived cell lines and cancer-associated fibroblasts

**Figure 5:**
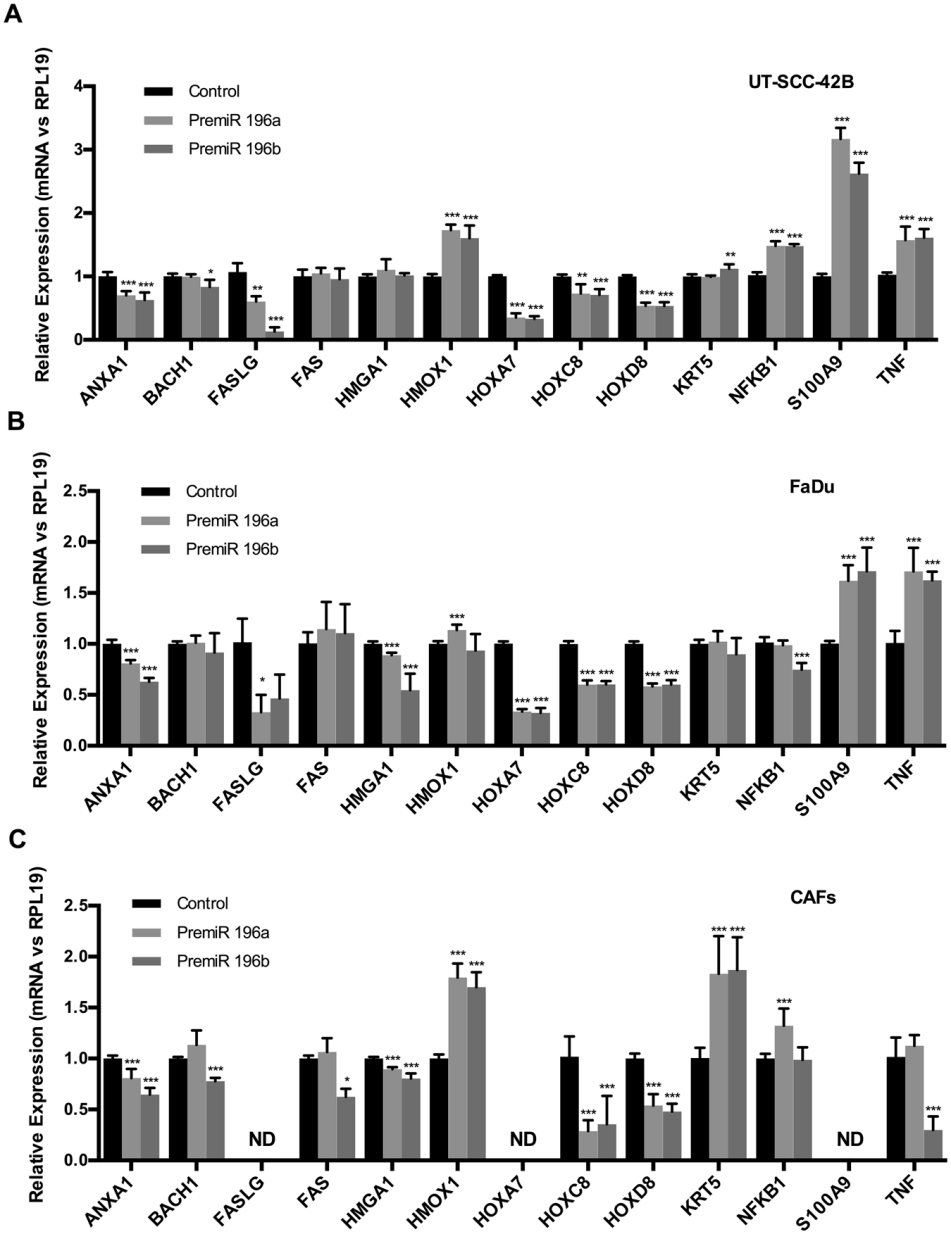
Analysis of miR-196a and miR-196b target genes in HNSCC cells and CAFs. mRNA expression levels of potential/validated miR-196a/b target genes were measured by RT-qPCR in (**A**) UT-SCC-42B, **(B**) FaDu and (**C**) CAFs after 72 h transfection with either premiR-196a, premiR-196b or non-targeting control. Data were normalized to RPL19 levels and relative to control-transfected cells. The graphs represent the mean ± SD, calculated from at least three independent experiments performed in triplicate. ND, not detected. *p < 0.05, **p < 0.01 and ***p < 0.001 by Student t-test.

The identification of putative miR-196a/b targets is of primary interest for deciphering their functional roles. Therefore, gene expression regulation by pre-miR-196a and pre-miR-196b transfection was examined in both HNSCC cells and CAFs on a panel of validated and/or predicted miRNA target genes in HNSCC and other cancer types (reviewed in^13,14^, and miRTarBase, Tarbase and TargetScan). Results showed that the endogenous levels of various genes (ANXA1 and HOX family) were consistently and significantly down-regulated upon miR-196a and miR-196b over-expression in UT-SCC-42B, FaDu and CAFs (Fig. 5), in accordance with their proposed mediator role as direct target genes. FASLG expression levels were significantly down-regulated by miR-196a and miR-196b in FaDu cells and also by miR-196a in UT-SCC-42B cells, but undetectable in CAFs. Hence, this study provides the first experimental evidence to validate FASLG gene as a miR-196a/b target gene in HNSCC. In contrast, the expression of certain other genes (HMOX1, KRT5, NFKB1, S100A9, TNF) was found to be significantly and differentially upregulated depending on the cell type, suggesting indirect regulation by miR-196a/b. We also observed several cell- and miRNA-specific changes, such as the downregulation of TNF and FAS expression by miR-196b in CAFs, or the downregulation of NFKB1 by miR-196b in FaDu cells. None of these genes have been previously demonstrated to be miR-196b target genes in HNSCC and CAFs.

### Distinctive effects of miR-196a and miR-196b expression on the activation of signaling pathways in HNSCC-derived cell lines

**Figure 6:**
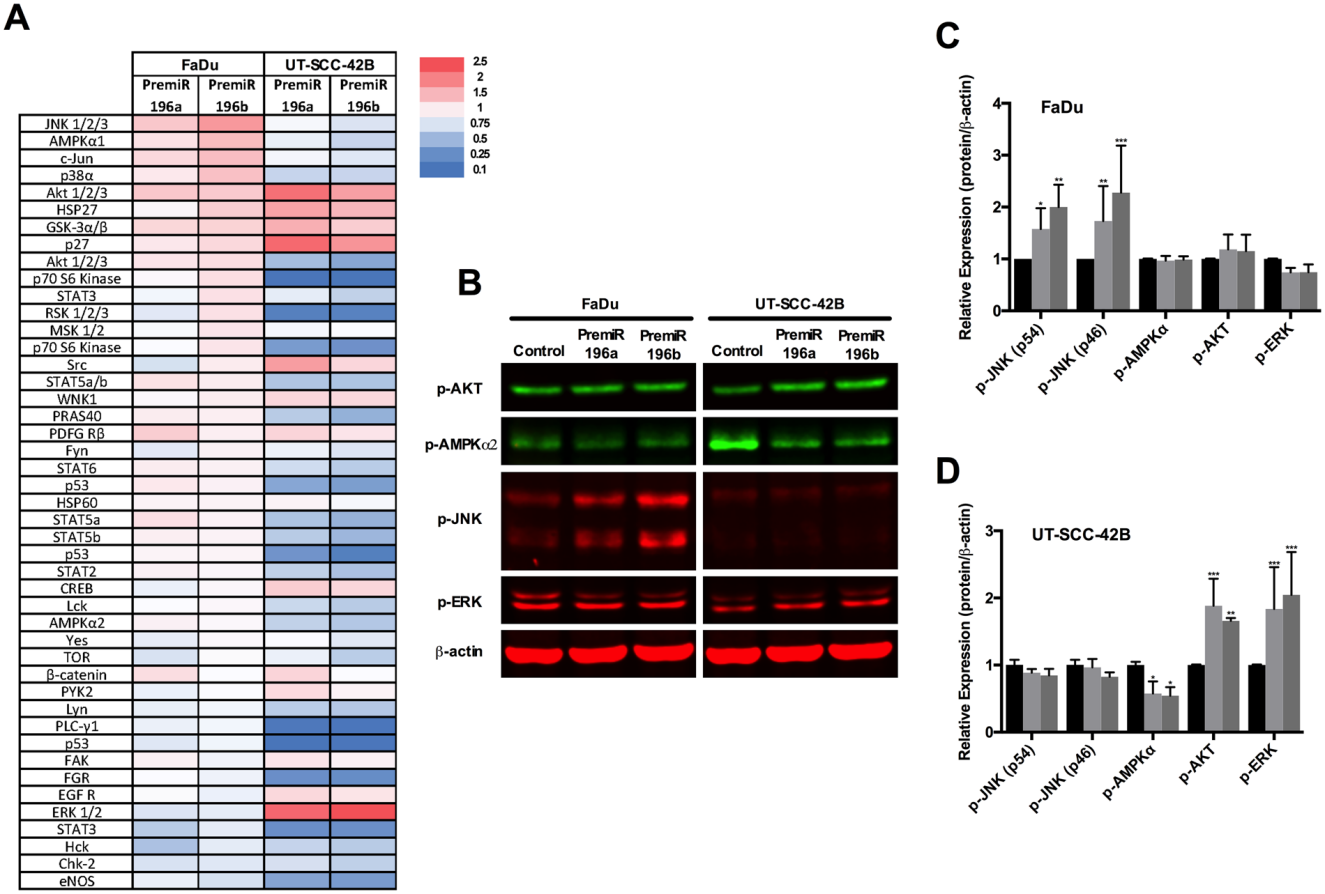
Analysis of signaling pathways targeted by miR-196a and miR-196b. Total protein lysates from UT-SCC-42B and FaDu cells transfected with either premiR-196a, premiR-196b or non-targeting control were applied to a proteome phospho-kinase array. (**A**) Relative signal intensity values (to control-transfected cells) are displayed as a heat map. (**B**) Western blot validation of the array data. (**C**) and (**D**) Quantification of IRDye fluorescent signals. *p < 0.05, **p < 0.01 and ***p < 0.001 by Student t-test.

The differential functional effects caused by miR-196a/b overexpression on UT-SCC-42B and FaDu cells were further investigated by analyzing the activation status of multiple intracellular signaling pathways, using a phosphokinase array. As shown in a heat map representation (Fig. 6A), miR-196a/b overexpression led to widespread changes in the phosphorylation levels of protein kinases. Increased phosphorylation was observed in FaDu cells, predominantly in JNK/c-Jun and p38 MAP kinases. This contrasted with an overall decrease in phosphorylation levels of various kinase cascades that regulate cell proliferation, as detected in UT-SCC-42B cells, such as JNK/c-Jun, p38 and MAPKs, PI3K/mTOR and STAT signaling pathways. The phosphorylation of certain sites was robustly and specifically induced in UT-SCC-42B cells by both miR-196a/b: SRC (Tyr419), AKT (Ser473), and ERK1/2 (Thr202/Tyr204; Thr185/Tyr187), although it is unlikely this would contribute substantially to the reduced proliferation observed in this cell line. A robust induction of p27 (Thr198) phosphorylation was also specifically detected in UT-SCC-42B, possibly involved in cell cycle control and regulation of cell growth.

The results obtained with the protein array were further validated by Western blot. Figure 6B–D confirms the cell-specific changes in phosphorylation detected in both HNSCC cell lines i.e. JNK (pT183/pY185) activation in FaDu cells, downregulation of phosphoAMPKα (Thr172) in UT-SCC-42B, and activation of pAKT (Ser473) and ERK (T185/Y187) in UT-SCC-42B cells.

## Discussion

miRNAs have emerged as key regulators of gene expression and crucial players in health and disease^8–10^. The characterization of miRNA expression patterns in cancers has demonstrated utility in disease diagnosis, prognosis, and therapy^31–34^. The frequent dysregulation of miR-196 family members in numerous cancer types reflects a close relationship with disease status and potential as biomarkers^13,14^. Nevertheless, the clinical and biological significance and underlying regulatory networks have been explored only to a limited extent, a prerequisite before any clinical application can be safely introduced. Although most studies have demonstrated prooncogenic functions of miR-196a/b in multiple cancer types^15–21^, there is contrary evidence of a tumor suppressive role^22–24^. The miR-196 family may have many different targets depending on the cellular and tissue context, and consequently, the ultimate impact on tumor pathogenesis will depend on the balance between oncogenes and tumor suppressor genes targeted by these miRNAs in specific cell types. This, in turn, is moderated by distinct promoter regulation for each of these miR-196′s and variant binding sites in their target genes.

The tumor and the surrounding microenvironment affect each other through constant physical and chemical interactions and together exert a major influence on tumor progression and disease outcome. miRNAs can be secreted into exosomes thereby acting as paracrine signaling factors between cancer cells and the tumor microenvironment (TME)^35,36^. Taking this into consideration, we initiated this study on the expression and functional role of miR-196b in both HNSCC and CAFs as a major component of tumor stroma. Interestingly, our study provides the first evidence for the functional and regulatory role of miR-196a/b in CAFs. Consequently, miR-196a/b may provide an important bidirectional communication between cancer cells and the surrounding CAFs. Nevertheless, miR-196 could also mediate paracrine communication with other TME components, such as immune or endothelial cells. Therefore, the functional impact of miR-196 may be even greater than herein described, which merits detailed investigation.

Quantitative analysis consistently showed increased levels of both miR-196a and miR-196b in an overwhelmingly high percentage (95–100%) of HNSCC tissue specimens. Expression varied in levels and percentage, being higher for miR-196a than miR-196b both in magnitude and frequency. In addition, increased levels of both miRNAs were detected in a considerable proportion (27–45%) of patient-matched normal epithelia, suggesting the early occurrence of these molecular changes. This hypothesis was further substantiated by analyzing miRNA expression in the early stages of HNSCC tumorigenesis. Thus, miR-196a and miR-196b expression was found markedly increased in 95–100% laryngeal precancerous lesions, and expression levels were maintained or even augmented in the patient-matched invasive carcinomas subsequently developed. Our findings also revealed that miR-196a/b expression did not reliably predict the risk of progression to invasive carcinoma. Consequently, although miR-196a and miR-196b dysregulation appears to be characteristic of HNSCC pathogenesis, it does not seem to play a determinant role in malignant transformation. miR-196a/b dysregulation could instead be a consequence of uncontrolled proliferation rather than a causative agent in tumor development and acquisition of the invasive phenotype. Similarly, miR-196a and miR-196b levels were not found to correlate significantly with the clinicopathologic characteristics of HNSCC patients, suggesting their application as tumor markers for diagnosis or disease monitoring rather than prognostic factors.

Since miRNAs are secreted into exosomes and stably released into the circulation, their detection in body fluids could provide novel non-invasive biomarkers for clinical application to replace or complement currently invasive biopsy approaches. Liquid biopsy has emerged as an alternate option to track disease changes in a simple, minimally invasive manner, allowing for serial sampling to inform about the tumor heterogeneity, response to treatment and/or minimal residual disease^37^. Saliva is an accessible biofluid surrounding HNSCC that can serve as a biomarker source with multiple advantages over blood sampling, such as easier and safer collection, handling and storage^38^. This study determined that increased levels of miR-196a/b can be detected in saliva samples from HNSCC patients, highlighting their potential utility as biomarkers for early HNSCC detection and/or disease surveillance in tissue samples and saliva. Similarly, elevated miR-196a/b levels have been detected in plasma from patients with digestive tract cancers^14,39^, thus broadening their clinical applicability as circulating tumor markers.

*In vitro* functional studies further characterized the pathobiological role of miR-196a/b dysregulation in both HNSCC-derived cell lines and CAFs. According to our results, miR-196a/b exhibit multifaceted roles in HNSCC and the surrounding tumor stroma. We found that both miR-196a and miR-196b contributed to cell proliferation and migration in tumor cells, although led to opposite effects depending on the HNSCC cell line tested (i.e. both pre-miR196a and miR196b consistently increased proliferation/migration in FaDu cells while decreased proliferation/migration in UT-SCC-42B). Thus, miR-196a and miR-196b were both pro-oncogenic in FaDu and suppressive in UT-SCC-42B cells. miR-196b also specifically inhibited the migratory and invasive potential of CAFs. These results clearly demonstrate that altered expression of miR-196a and miR-196b causes pleiotropic effects in HNSCC depending on specific cellular and tissue contexts.

The possible molecular mechanisms whereby miR-196a/b regulates cellular functions were investigated to identify potential gene targets and downstream regulatory pathways, including ANXA1 and HOX genes, commonly regulated by miR-196a/b. This study provided original experimental evidence for several novel genes targeted by miR-196a/b in HNSCC cells, such as FASLG and NFKB1, or TNF and FAS specifically targeted by miR-196b in CAFs. Despite the single nucleotide difference between mature miR-196a and miR-196b, these two molecules have shown a wide range of expression and functional roles in different cancers. Thus, both miR-196a/b targeted NME4 expression to induce cell migration and invasion in oral cancer^15^. miR-196a was found to enhance radioresistance in HNSCC cells by suppressing ANXA1^16^. In gastric cancer, miR-196a regulated the cell cycle regulator p27kip1 to promote cell proliferation^17^. miR-196a also promoted cancer progression by targeting NFκB-Iα in pancreatic cancer^19^. In colorectal cancer, miR-196a inhibited the expression of HOXA7, HOXB8, HOXC8 and HOXD8 genes, leading to AKT pathway activation and increased cell motility^40^. Also in colorectal cancer, miR-196b repressed FAS expression modulating cell apoptosis^24^. miR-196a promoted cell proliferation and invasion mediated by HOXA5 in lung cancer^21^. miR-196a was also found to promote cell proliferation and migration by inhibiting p27kip1, FOXO1 or NTN4 in cervical cancer^20,41^. In marked contrast, miR-196a inhibited HOXC8 to reduce cell proliferation and invasion in melanoma^26^. miR-196 was also found to suppress cell invasion in breast cancer, although miR-196 expression levels did not correlate with clinical metastasis status^27^. In chronic myeloid leukemia, low miR-196b levels enhanced the activity of BCR-ABL1 and HOXA9 oncogenes^28^.

We also found that miR-196a/b caused widespread changes in the activation status of multiple intracellular signaling pathways in a cell-dependent manner. Noteworthy, the proteome arrays (Fig. 6A) showed consistent phosphorylation changes by miR196a and miR196b on FaDu cells, although dramatically different to those in UT-SCC-42B cells. These data suggest that the distinct cell/tissue specificity among the various regulatory targets of miR-196a/b and ultimately the dynamic balance between the oncogenes and tumor suppressors targeted determine cell fate. This reflects the complexity of the miRNA-target regulatory network and how a single miRNA may act as oncomir or antagomir depending on specific cell/tissue context. Due caution must be taken when planning to target a miRNA for cancer therapy.

Together, these findings reveal the early occurrence and prevalence of miR-196b dysregulation in HNSCC tumorigenesis, suggesting its possible utility for early diagnosis and/or monitoring of disease status. Furthermore, the potential of mir-196a/b as non-invasive biomarkers in saliva was also considered. However, the pleiotropic effects caused by miR-196a/b in HNSCC and the surrounding microenvironment cast doubt on their possible application as therapeutic targets.

## Methods

### Patients and Tissue Specimens

Surgical tissue specimens (fresh frozen) from 19 patients with HNSCC who underwent resection of their tumors at the Hospital Universitario Central de Asturias between 2000 and 2002 were collected, in accordance with approved institutional review board guidelines. All experimental protocols were approved by the Institutional Ethics Committee of the Hospital Universitario Central de Asturias and by the Regional CEIC from Principado de Asturias (approval number: 70/16 for the project PI16/00280). Informed consent was obtained from all patients. All patients had a single primary tumor (in the larynx or pharynx), microscopically clear surgical margins and received no treatment prior to surgery. The clinicopathologic characteristics of these patients are shown in Supplementary Table S1. Patient-matched normal adjacent epithelia were also collected from the same anatomical site (contralateral) in 11 out of 19 patients. In addition, normal epithelia from non-oncologic patients without exposure to tobacco carcinogens (i.e. childhood tonsillectomy) were collected and used as healthy controls.

Formalin-fixed paraffin-embedded (FFPE) tissue samples from 40 patients who were diagnosed of laryngeal dysplasia at the Hospital Universitario Central de Asturias and the Hospital Universitario Donostia were retrospectively collected, in accordance with approved institutional review board guidelines. The histological diagnosis was confirmed by an experienced pathologist. The clinical features of these patients are summarized in Supplementary Table S2. Tumor blocks were also obtained from those patients who developed an invasive carcinoma (23 cases). All patients were treated by excisional biopsy using stripping microflap excision with cold instruments. A complete macroscopic exeresis of the lesion was performed in all cases, but the microscopic margins were not addressed. Patients were followed-up for a minimum of 60 months or until progression to malignancy occurred. FFPE normal epithelium from 5 patients was also collected and used as control.

Saliva samples were prospectively collected from 15 HNSCC patients (13 male and 2 female, age range 46–88 years) before surgical treatment of their tumors at the Hospital Universitario Central de Asturias, in accordance with the Ethics Committee approval. All patients had a single primary tumor, microscopically clear surgical margins and received no treatment prior to surgery. Saliva samples from 11 healthy donors (8 male and 3 female, age range 26–71 years) were also collected. Saliva samples were collected following the procedure previously described^42^, and frozen at −80 °C until miRNA purification. The entire volume of saliva sample, without centrifugation of cells, was used for miRNA purification.

### miRNA expression analysis

Total RNA (including miRNA) was extracted from fresh frozen tissue and/or cultured cells using Trizol (Invitrogen Life Technologies). miRNAs were isolated from FFPE tissue samples (both laryngeal precancerous lesions and patient-derived invasive tumors subsequently developed) using the RecoverAll kit from Ambion (Life Technologies, Paisley, UK), according to the manufacturer’s instructions. FFPE tissue sections from 40 laryngeal precancerous lesions (5–10 slides of 10 µm thickness) were selected to ensure >50% dysplastic cells. For patient-derived invasive tumors, two punches of 2 mm diameter were taken from representative areas of each FFPE tumor block using a new, sterile punch (Kai Europe GmbH, Solingen, Germany) to avoid cross-contamination. miRNAs from saliva samples were isolated using Nucleospin miRNA (Macharey Nagel). The relative levels of miR-196a and miR-196b were determined by two-step RT-PCR using miRNA-specific primers and probes according to the Taqman miRNA Assay protocol (Applied Biosystems) on a StepOnePlus Real-Time PCR System (Applied Biosystems). Reactions were run in triplicate, using RNU48 as control. The relative miRNA expression was calculated using the 2^−ΔΔ*C*T^ method and the data were normalized to RNU48 levels.

### Analysis of miRNA targets by real-time RT-PCR

Total RNA was extracted using Trizol reagent and cDNA synthesized with Superscript II RT-PCR System (Invitrogen Life Technologies), according to manufacturer’s protocols. Gene expression was analyzed by Real-time PCR using the StepOnePlus Real-Time PCR System (Applied Biosystems) following Applied Biosystems’ SYBR Green Master Mix protocol, using the primers detailed in Supplementary Table S3. The constitutively expressed RPL19 ribosomal coding gene was used as endogenous control. The relative mRNA expression was calculated using the 2^−ΔΔ*C*T^ method and the data were expressed as the fold-change normalized to RPL19 mRNA levels and relative to control-transfected cells.

### Cell Culture

FaDu cells (male, hypopharyngeal squamous cell carcinoma) were purchased to the American Type Culture Collection, and the HNSCC cell line UT-SCC-42B derived from a laryngeal squamous carcinoma (male, T4N3M0) was kindly provided by Dr. R. Grenman (Department of Otolaryngology, University Central Hospital, Turku, Finland)^43^. Cells were grown in DMEM supplemented with 10% fetal bovine serum (FBS), 100 U/ml penicillin, 200 mg/ml streptomycin, 2 mmol/l L-glutamine, 20 mmol/l HEPES (pH 7.3) and 100 mmol/l non-essential amino acids.

Cancer-associated fibroblasts (CAFs) were obtained from minced tumor tissue of surgically resected HNSCC (male, hypopharyngeal squamous cell carcinoma, T2N1M0) at the Hospital Universitario Central de Asturias. Briefly, fresh tumor samples from the operating theatre were cut into several small fragments, transferred to dry 25 cm^2^ culture flasks and covered with a drop of complete culture medium (DMEM supplemented with 10% FBS, 100 U/ml penicillin, 200 mg/ml streptomycin, 2 mmol/l L-glutamine, 20 mmol/l HEPES (pH 7.3) and 100 mmol/l non-essential amino acids) and incubated in 5% CO_2_ at 37 °C. Initial outgrowth of both tumor and fibroblast cells was observed over 5–7 days, and fibroblasts were collected by repeated selective trypsinizations. Isolated CAFs were further characterized by Vimentin and α-SMA staining. All the cells used derived from HPV-negative primary tumors. Cell line authentication was performed by DNA (STR) profiling at the SCT Core Facilities (University of Oviedo, Asturias, Spain). All cell lines were tested periodically for mycoplasma contamination by PCR to specifically amplify a conserved region of the mycoplasma 16 S RNA gene (Biotools Detection kit).

### Transfections with pre-miR miRNA precursors

Ambion Pre-miR miRNA Precursors AM17100 hsa-miR-196a (#PM10068) and hsa-miR-196b (#PM12946) were purchased to Thermo Fisher Scientific and siGLO RISC-Free™ was used as non-targeting control (Thermo Scientific Dharmacon).

HNSCC cells and CAFs were seeded in 6-well plates at a density of 60,000 and 90,000 cells, respectively. The next day, cells were transfected with 100 nM of either pre-miR-196a or pre-miR-196b or a non-targeting control using Lipofectamine 2000 Transfection Reagent (Invitrogen Life Technologies), according to the manufacturer’s protocol. miRNA expression or mRNA gene expression was tested by real-time RT-PCR at 72 h post-transfection.

### Proteome Array and Western blot Analysis

Proteins were extracted from HNSCC cells transfected with either pre-miR-196a, pre-miR-196b or a non-targeting control at 72 h post-transfection using lysis buffer (R&D Systems). 600 μg of each cell lysate was applied to the Proteome Profiler Human Phospho-Kinase Array (ARY003; R&D Systems, Minneapolis, MN) according to the manufacturer’s instructions. This is a membrane-based antibody array that allows simultaneous determination of the relative site-specific phosphorylation levels of 43 human protein kinases. A cocktail of biotinylated detection antibodies, streptavidin–horseradish peroxidase and chemiluminescent detection reagents were used to detect the phosphorylated protein. Membranes were scanned with the Odyssey Fc Dual-Mode Imaging System (LI-COR Biosciences), and signal density analysis was performed using Image Studio Lite software (LI-COR Biosciences).

Validation of specific phosphorylated proteins was carried out by Western blot analysis. Thus, protein lysates were resolved by SDS–polyacrylamide gel electrophoresis (SDS–PAGE) and transferred subsequently to nitrocellulose membranes (Amersham Protran, GE Healthcare).

The membranes were blocked for 1 h with Odyssey blocking buffer and incubated overnight with the following specific primary antibodies at 1:1,000 dilution: JNK (pT183/pY185) (BD Biosciences #612540), phospho-Akt (Ser473) (Cell Signaling #9271), phospho-AMPKα (Thr172) (40H9) rabbit mAb (Cell Signaling #2535), ERK2 (T185/Y187) Monoclonal Antibody (R&D Systems #MAB1825), and β-actin (dilution 1:10,000 for 1 h; Sigma Aldrich #AC15). The IRDye Infrared Fluorescent secondary antibodies Goat anti-Rabbit IRDye 800CW and Goat anti-Mouse IRDye 680RD were used for detection. Membranes were scanned with the Odyssey Fc Dual-Mode Imaging System (LI-COR Biosciences) using the red (700 nm) and green (800 nm) channels. Uncropped images of all the immunoblots can be found in Supplementary Information.

### Cell proliferation assays

HNSCC cells and CAFs were respectively seeded into 96-well plates at a density of 1,500 and 3,000 cells *per* well, and cell proliferation was measured after 4 days. Quantification of cell number was determined in quadruplicates using a tetrazolium-based MTS test (CellTiter 96 Aqueous One Solution Cell Proliferation Assay from Promega, Madison, WI) reading the absorbance at 490 nm with the use of a Synergy HT plate reader (BioTek, Winooski, VT).

### Scratch-Induced Directional Migration Assay

Cells were plated in 24-well tissue culture dishes with ibidi® culture inserts (ibidi LLC, Verona, WI, USA) at 80–90% confluence. The following day, the culture inserts were detached to form a cell-free gap in the cell monolayer and the medium was changed to remove floating cellular debris. Migration was monitored using a Zeiss Cell Observer Live Imaging microscope (Zeiss, Thornwood, NY) coupled with a CO_2_ and temperature-maintenance system. Time-lapse images were acquired every 30 min during 24 h using a Zeiss AxioCam MRc camera. The area of each individual wound gap was measured in each culture condition using Image J image analysis program. Cell migration was analyzed by calculating the difference between the initial scratch area and the residual gap after 5, 15 or 20 h for UT-SCC-42B, FaDu or CAFs, respectively. For each culture condition, cell migration was quantified by comparing the area of the cell-free gap after normalization to the control cells.

### Collective three-dimensional spheroid invasion assays

Cells were suspended in DMEM plus 5% Methyl cellulose (Sigma) at 80,000 cells/ml medium to form cell spheroids (2,000 cells/spheroid) by serial pipetting of 25 μl into a non-adhesive Petri dish, and incubated in an inverted position for 18 h. Next day, each cell spheroid was transferred to an individual well of 96-well plate and embedded into bovine collagen matrix (Advanced Biomatrix PureCol), and filled with 100 μl of complete media. Collective cell invasion was monitored using a Zeiss Cell Observer Live Imaging microscope (Zeiss, Thornwood, NY) coupled with a CO_2_ and temperature-maintenance system. Time-lapse images were acquired every 30 min during 20 h using a Zeiss AxioCam MRc camera. The invasive area was determined by calculating the difference between the final area (t = 20 h) and the initial area (t = 0) using image J analysis program, and data were normalized to the control cells.

### Statistical Analysis

The data are presented as the mean ± standard deviation (SD) unless otherwise stated, and compared using one-way ANOVA or unpaired Student’s t-test. Statistical analysis was performed using GraphPad Prism version 6.0 (Graphpad Software Inc, La Jolla, CA, USA). *p* values less than 0.05 were considered statistically significant (*p* < 0.05, *p < 0.01, **p < 0.005, ***).

## Electronic supplementary material

Supplementary Information
