# Supplementary material for: Dysregulation of Mir-196b in Head and Neck Cancers Leads to Pleiotropic Effects in the Tumor Cells and Surrounding Stromal Fibroblasts
Source: Sci Rep. 2017 Dec 19;7:17785. doi: 10.1038/s41598-017-18138-8 (PMC5736577; doi:10.1038/s41598-017-18138-8)
Supplement: Supplementary file 1 — Supplementary Information [file 41598_2017_18138_MOESM1_ESM.pdf]

SUPPLEMENTARY INFORMATION

TITLE: DYSREGULATION OF miR-196b IN HEAD AND NECK CANCERS LEADS TO PLEIOTROPIC EFFECTS IN THE TUMOR CELLS AND SURROUNDING STROMAL FIBROBLASTS

AUTHORS:

Saúl Álvarez-Teijeiro[1], Sofía T. Menéndez[1], M. Ángeles Villaronga[1], Juan P. Rodrigo[1], Lorea Manterola[2], Lucas de Villalaín[3], Juan C. de Vicente[3], Laura Alonso-Durán[1], M. Pilar Fernández [4], Charles H. Lawrie [2,5], Juana M. García-Pedrero [1*].

[1] Department of Otolaryngology, Hospital Universitario Central de Asturias and Instituto de Investigación Sanitaria del Principado de Asturias, IUOPA, University of Oviedo, Oviedo, CIBERONC, Spain.

[2] Molecular Oncology Group, Biodonostia Research Institute, San Sebastián, Spain.

[3] Department of Oral and Maxillofacial Surgery, Hospital Universitario Central de Asturias and Instituto de Investigación Sanitaria del Principado de Asturias, IUOPA, University of Oviedo, Oviedo, Spain.

[4] Department of Biochemistry and Molecular Biology, University of Oviedo, Oviedo, Spain.

[5] IKERBASQUE, Basque Foundation for Science, Bilbao, Spain

**** Correspondence:***

Juana María García-Pedrero, PhD E-mail: juanagp.finba@gmail.com

Juan Pablo Rodrigo, MD, PhD E-mail: juanpablo.rodrigo@sespa.es

Hospital Universitario Central de Asturias

Edificio FINBA Laboratorio ORL – IUOPA

Avda Roma s/n 33011 Oviedo, Spain

Phone: +34 985107937

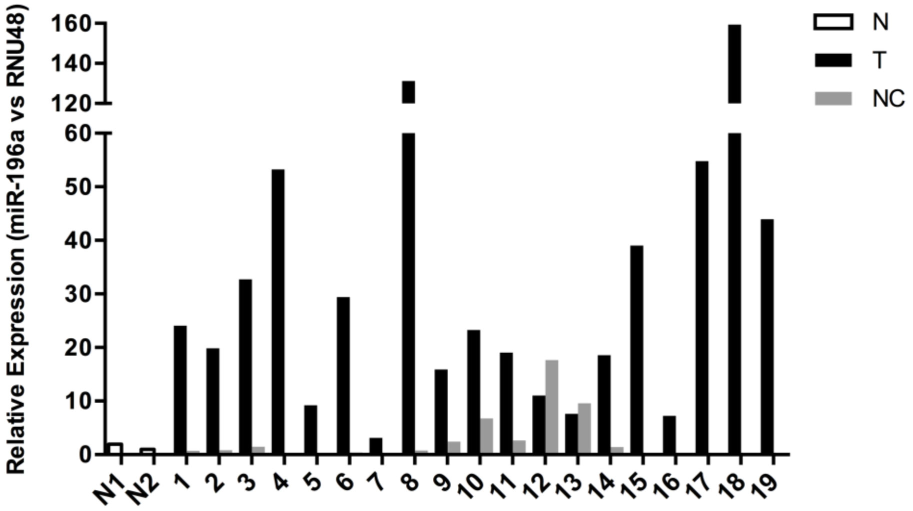

Supplementary Figure S1

Analysis of miR-196a expression in HNSCC tissue specimens. miR-196a expression levels were quantified by RT-qPCR in 19 fresh primary tumors (T) and patient-matched normal counterparts (NC). Data were normalized to RNU48 levels, and relative to the normal mucosa from non-oncologic patients (N).

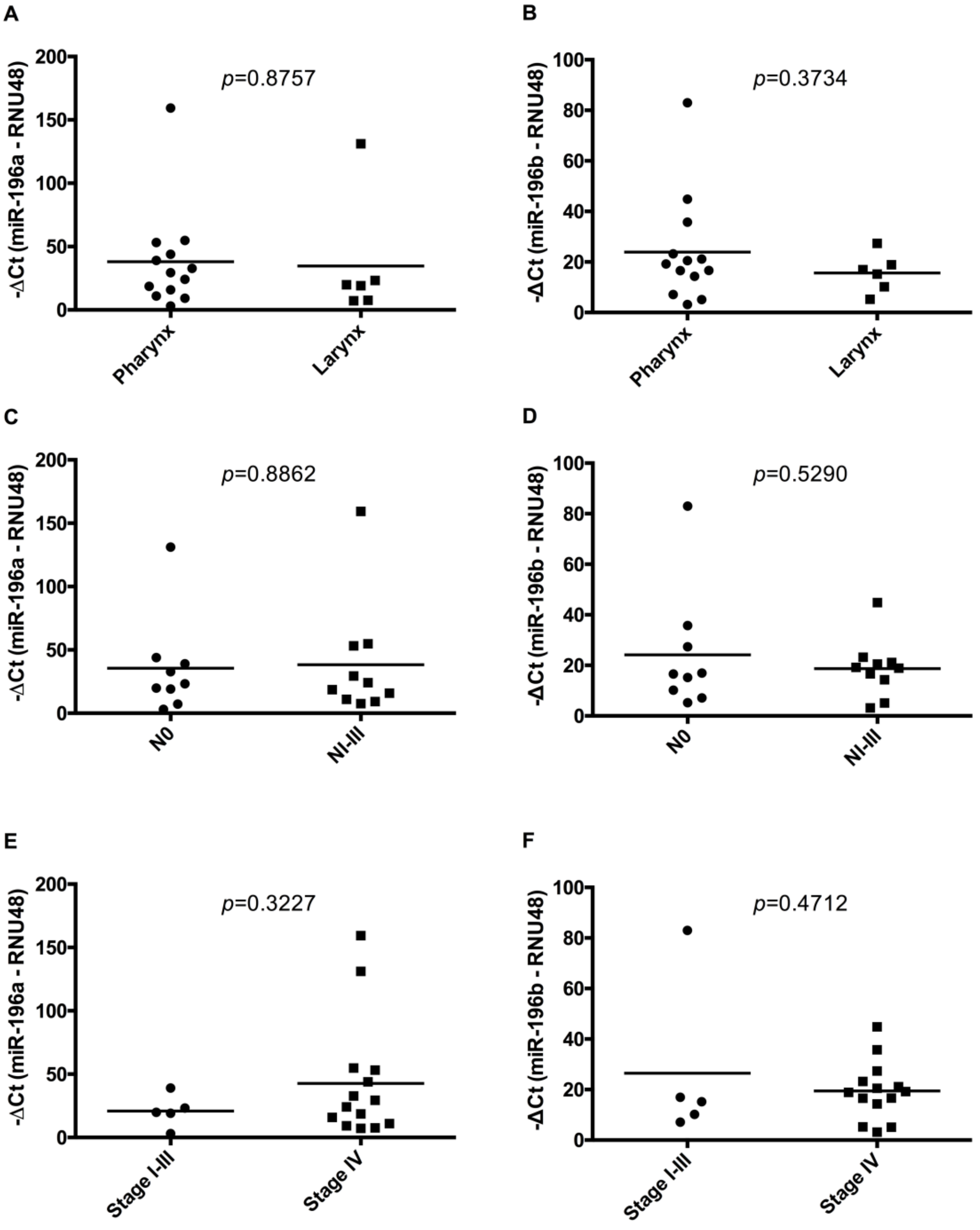

Supplementary Figure S2

miR-196a and miR-196b levels in HNSCC tissue specimens distributed according to the tumor site **(A and B)**, pN classification **(C and D)**, and disease stage **(E and F)**. *p* values calculated by Student t-test.

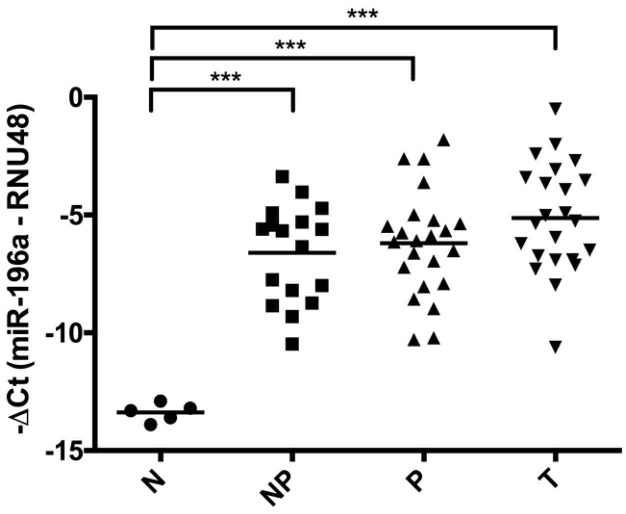

Supplementary Figure S3

Analysis of miR-196a expression in early stages of HNSCC tumorigenesis. miR-196a expression levels were quantified by RT-qPCR in 40 patients with laryngeal precancerous lesions, comprising 17 non-progressing dysplasias (NP), 23 progressing dysplasias (P) and the 23 patient-matched invasive tumors (T) subsequently developed. Normal epithelia from 5 non-oncologic patients were included as (N) healthy controls. ****p* < 0.001 by Holm-Sidak's multiple comparisons test.

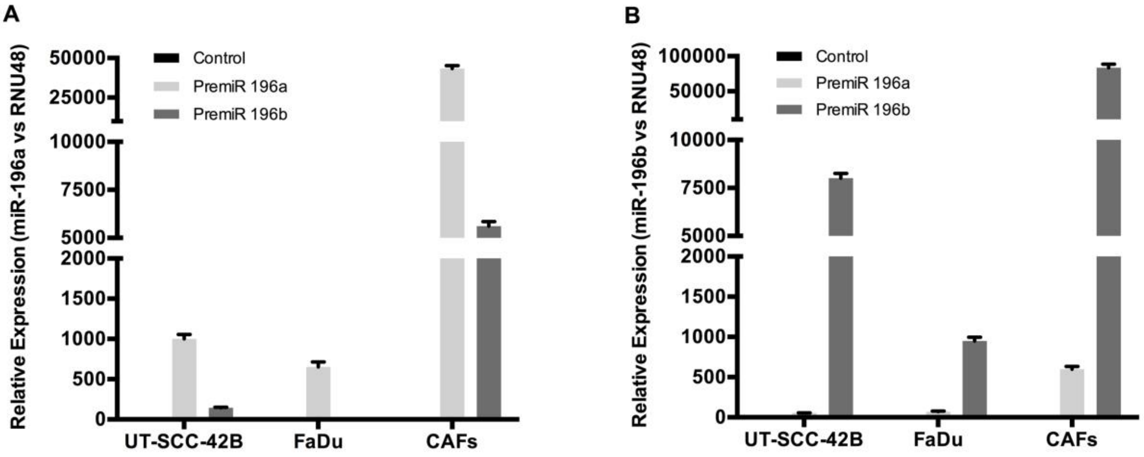

Supplementary Figure S4

Analysis of miR-196a and miR-196b expression in HNSCC cells and CAFs transfected with premiR precursors. **(A)** miR-196a and **(B)** miR-196b expression levels were quantified by qRT-PCR in UT-SCC-42B, FaDu or CAFs transfected with either premiR-196a, premiR-196b or non-targeting control. Data were normalized to RNU48 levels and relative to control-transfected cells. The graphs represent the mean ± SD of at least three independent experiments run in triplicate.

Supplementary Table S1. Clinicopathologic characteristics of the HNSCC patients (N=19)

Characteristic	No. (%)
**- Mean age, years**	60
**- Gender**	
Male	17 (89)
Female	2 (11)
**- pT classification**	
T1-T2	5 (26)
T3	5 (26)
T4	9 (47)
**- pN classification**	
N0	9 (47)
N1-3	10 (53)
**- Disease stage**	
I-II	3
III	2
IV	14
**- Pathological grade**	
Well differentiated	9 (47)
Moderately differentiated	3 (16)
Poorly differentiated	7 (37)
**- Site**	
Pharynx	13 (68)
Larynx	6 (32)
**Total Cases**	19

Supplementary Table S2. Clinicopathologic characteristics of the premalignant patients (N=40)

Characteristic	No. (%)
**- Mean age, years**	65
**- Gender**	
Male	36 (90)
Female	4 (10)
**- Grade classification**	
Low-grade	17 (42)
High-grade	23 (58)
**Total Cases**	40

Supplementary Table S3

Primers used for real-time RT-PCR (5’ → 3’)

ANXA1-Fw	GCAGGCCTGGTTTATTGAAA
ANXA1-Rv	GCTGTGCATTGTTTCGCTTA
BACH1-Fw	CTGATGGAGAGCTGAACATTACTCTT
BACH1-Rv	AGCAGTGTAGGCAAACTGAATTAAAG
FASLG-Fw	AGAGTCTACCAGCCAGAT
FASLG-Rv	AGTTGGACTTGCCTGTTA
FAS-Fw	ATGGCCAATTCTGCCATAAG
FAS-Rv	GACAAAGCCACCCCAAGTTA
HMGA1-Fw	ACAGCGCTGGTAGGGAGTCA
HMGA1-Rv	CCCCGAGGTCTCTTAGGTGTT
HMOX1-Fw	TGACCCGAGACGGCTTCA
HMOX1-Rv	CCTCCAGGGCCACATAGATG
HOXA7-Fw	TACCCCTGGATGCGGTCTT
HOXA7-Rv	CAGGTAGCGGTTGAAGTGGAA
HOXC8-Fw	TCCCAGCCTCATGTTTCCAT
HOXC8-Rv	TCTGATACCGGCTGTAAGTTTGC
HOXD8-Fw	CTCGTCTCCTTCTCAAATGTTTCC
HOXD8-Rv	GACTGTAGGTTTGTCTTCCTCTTCGT
KRT5-Fw	CCGCAGCCGGACAGAA
KRT5-Rv	TGTCTGCTGCAGCTCCTCAT
NFKB1-Fw	TGCCAACAGATGGCCCATAC
NFKB1-Rv	TGTTCTTTTCACTAGAGGCACCA
RPL19-Fw	GCGGAAGGGTACAGCCAAT
RPL19-Rv	GCAGCCGGCGCAAA
S100A9-Fw	TGTGGCTCCTCGGCTTTG
S100A9-Rv	GCGTTCCAGCTGCGACAT
TNF-Fw	CCAGGCAGTCAGATCATCTTCTC
TNF-Rv	AGCTGGTTATCTCTCAGCTCCAC

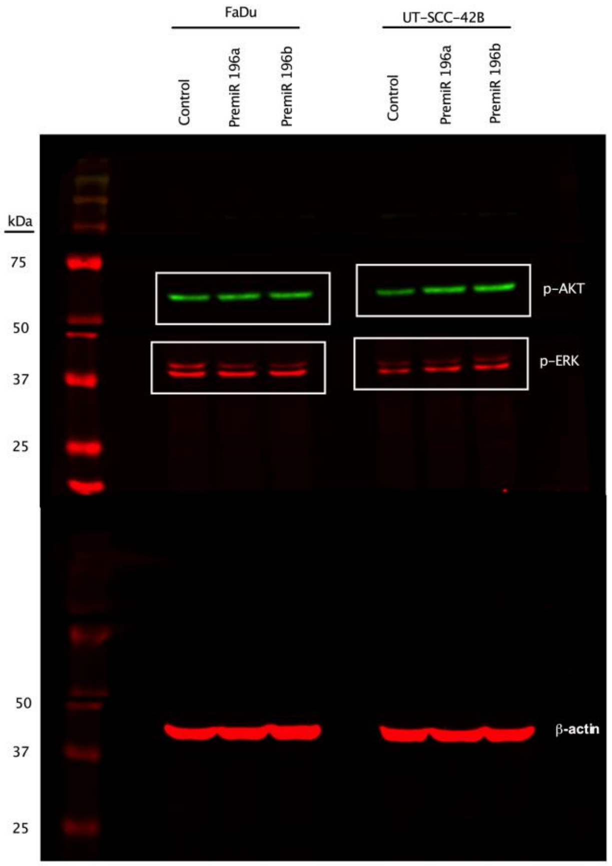

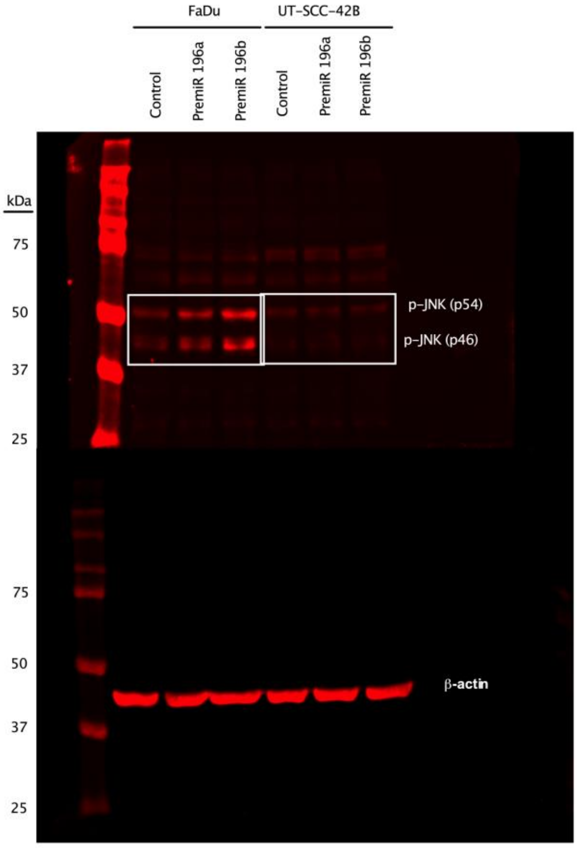

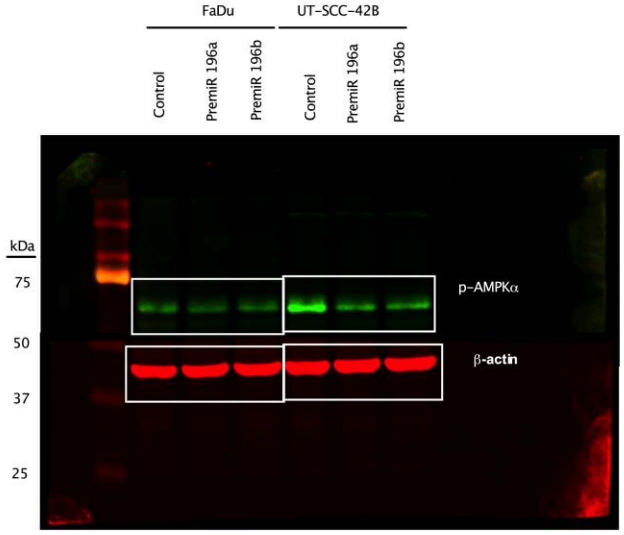

Uncropped membranes for Figure 6b
